# *Fantosmium*, a New Genus of Onthophilinae From Mid‐Cretaceous Kachin Amber (Coleoptera: Histeridae)

**DOI:** 10.1002/ece3.71849

**Published:** 2025-09-08

**Authors:** Yan‐Da Li, Di‐Ying Huang, Chen‐Yang Cai

**Affiliations:** ^1^ State Key Laboratory of Palaeobiology and Stratigraphy, Nanjing Institute of Geology and Palaeontology Chinese Academy of Sciences Nanjing China; ^2^ Bristol Palaeobiology Group, School of Earth Sciences University of Bristol Bristol UK

**Keywords:** fossil, Histeridae, Kachin amber, Mesozoic, parsimony, phylogenetic analysis

## Abstract

We describe and illustrate *Fantosmium qizhihaoi* gen. et sp. nov., a new histerid beetle from mid‐Cretaceous Kachin amber, and evaluate its phylogenetic position based on morphological characters. Our analysis recovers *Fantosmium* as part of the extant subfamily Onthophilinae, allied to the Cretaceous genera *Cretonthophilus* Caterino et al., *Phasmister* Caterino, and *Carinumerus* Caterino. It resembles these fossil genera in possessing ventrally open hypomeral antennal cavities and a projecting prosternal lobe. *Fantosmium* is particularly distinctive in its elytral morphology, exhibiting weakly elevated elytral carinae ornamented with small denticles, an intercarinal surface bearing irregular reticulate sculpture, explanate and irregularly punctate epipleura, and a strongly undulate posterior elytral margin. The discovery of *Fantosmium* provides new insights into the early evolutionary history and morphological diversity of Onthophilinae.

## Graphical abstract

We describe and illustrate a new onthophiline clown beetle from mid‐Cretaceous Kachin amber, *Fantosmium qizhihaoi* gen. et sp. nov. *Fantosmium* is particularly distinctive in its elytral morphology, providing new insights into the early morphological diversity of Histeridae.

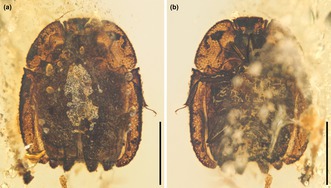

## 1 Introduction

The family Histeridae (clown beetles) represents a diverse and ecologically significant group of Coleoptera, with the earliest fossil dated to the Early Cretaceous (Zhou et al. 2020). Notably, mid‐Cretaceous Kachin amber from northern Myanmar has yielded the highest diversity of histerid fossil genera and species, including numerous novel taxa critical for understanding the group's early radiation (e.g., Caterino and Maddison 2018; Zhou et al. 2019; Jiang et al. 2022; Yamamoto and Caterino 2023; Simon‐Pražák et al. 2023, 2024, 2025). These exceptionally preserved fossils provide critical evidence for reconstructing both morphological transitions and ecological adaptations during the early evolution of this family.

Within this context, the subfamily Onthophilinae has attracted particular attention due to its taxonomically contested status and rich fossil representation in Kachin amber. Extant Onthophilinae exhibit marked morphological heterogeneity among constituent genera, with conflicting interpretations of subfamily boundaries by different taxonomists (e.g., Kovarik 1994; Ślipinśki and Mazur 1999; Bousquet and Laplante 2006; Kovarik and Caterino 2016). Notably, a recent molecular phylogeny, though with limited gene and taxon sampling, suggested that the current concept of Onthophilinae (i.e., *sensu* Kovarik and Caterino 2016) might not be monophyletic (Zhou et al. 2020). Compounding these challenges, Cretaceous fossil genera assigned to Onthophilinae exhibit some unusual traits such as ventrally open antennal cavity and antennal club divided by distinct annuli, further complicating subfamily‐level diagnoses (Caterino and Maddison 2018; Jiang et al. 2020; Caterino 2021; Caterino and Yamamoto 2023; Simon‐Pražák et al. 2024).

In the present study, we describe a remarkable new histerid genus from Kachin amber, which shows affinity with other Cretaceous Onthophilinae. This discovery expands the known morphological diversity of Cretaceous Onthophilinae, particularly in elytral morphology, and underscores the taxonomic richness of this subfamily prior to the Cenozoic.

## 2 Material and Methods

### 2.1 Material

**FIGURE 1:**
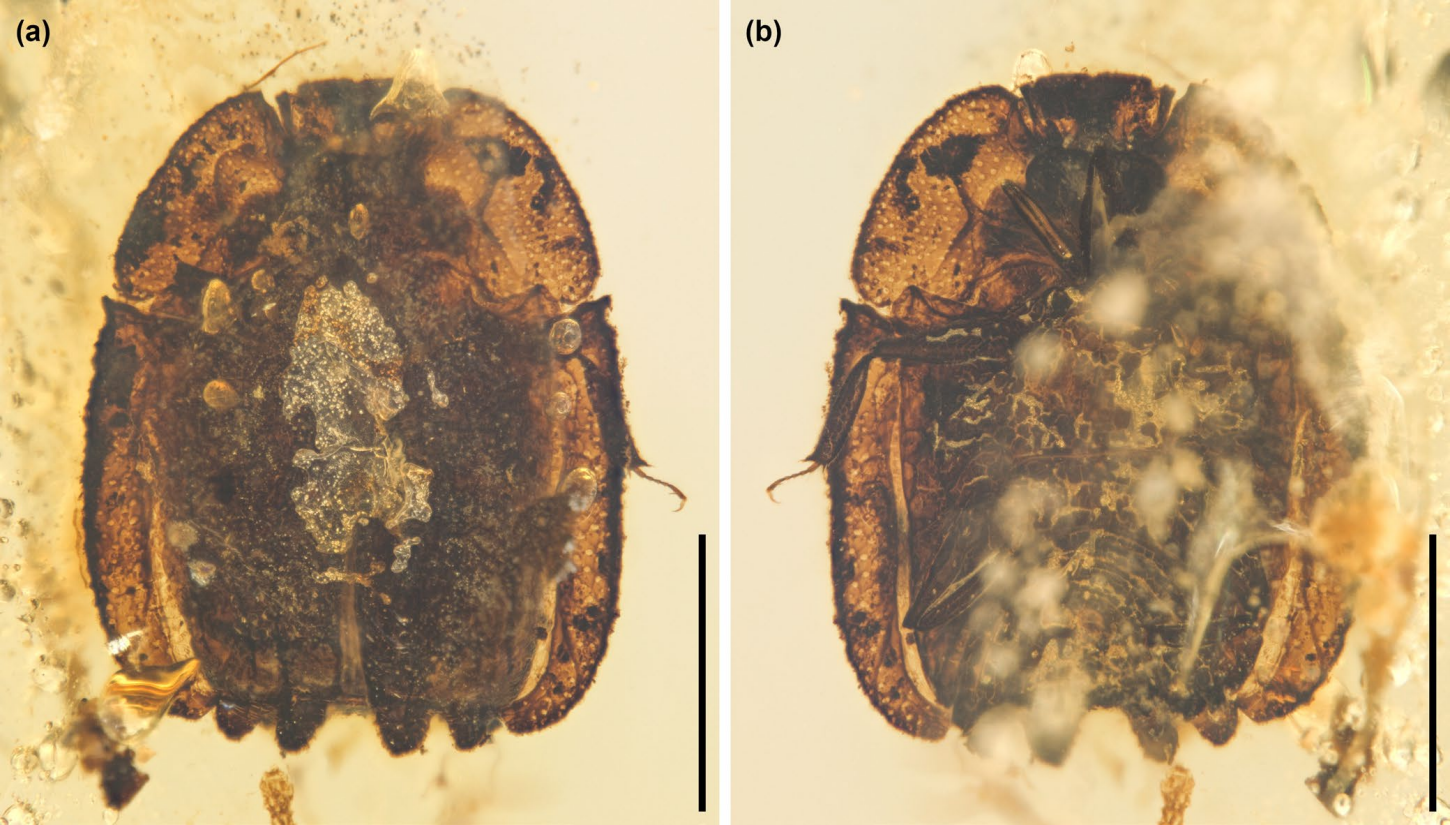
General habitus of *Fantosmium qizhihaoi*, holotype, NIGP207347, under reflected illumination. (a) Dorsal view. (b) Ventral view. Scale bars: 1 mm.

**FIGURE 2:**
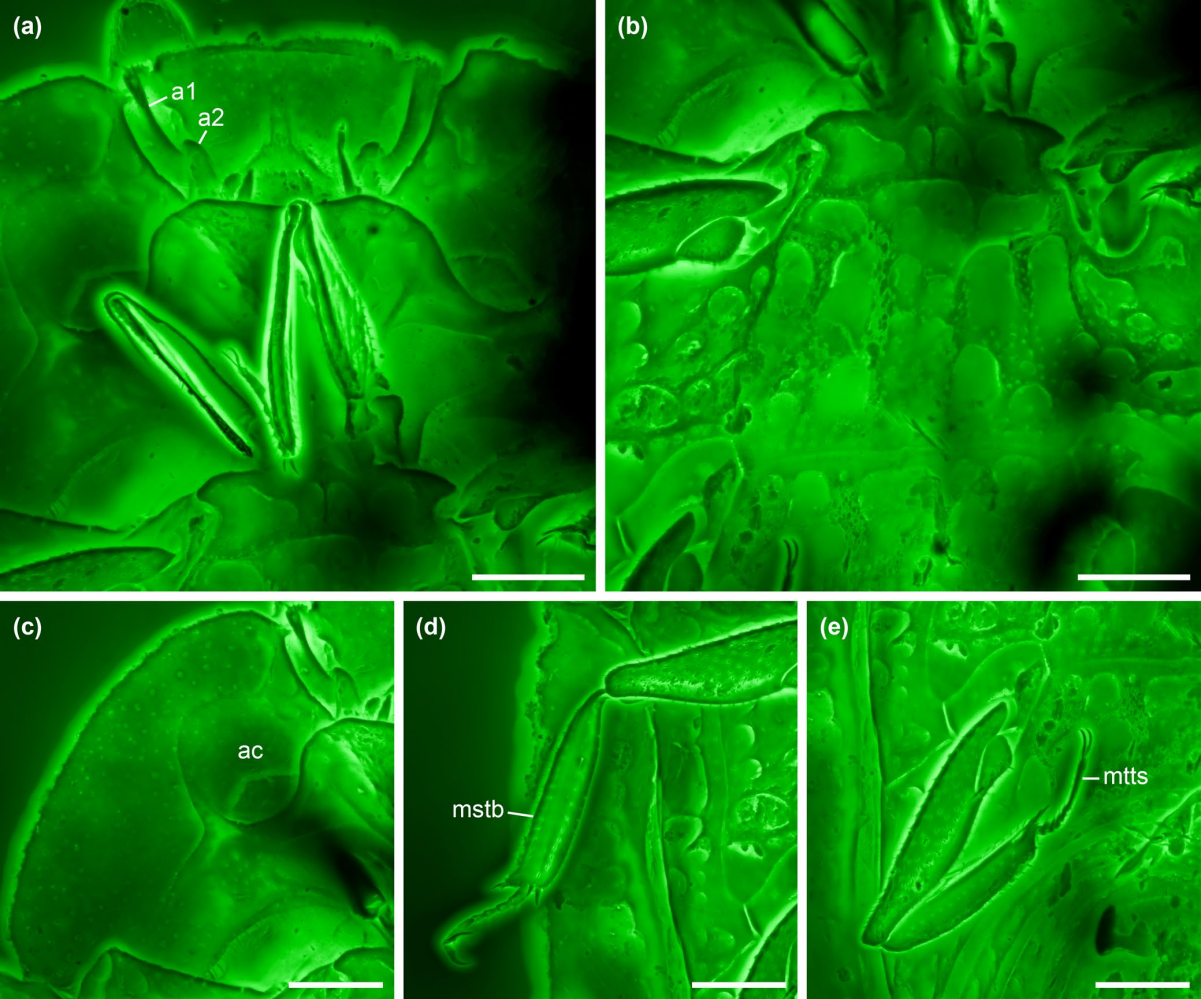
Details of *Fantosmium qizhihaoi*, holotype, NIGP207347, under confocal microscopy. (a) Head and prothorax, ventral view. (b) Meso‐ and metathorax, ventral view. (c) Hypomeron, ventral view. (d) Mid leg. (e) Hind leg. a1–2, antennomeres 1–2; ac, antennal cavity; mstb, mesotibia; mtts, metatarsus. Scale bars: 200 μm.

**FIGURE 3:**
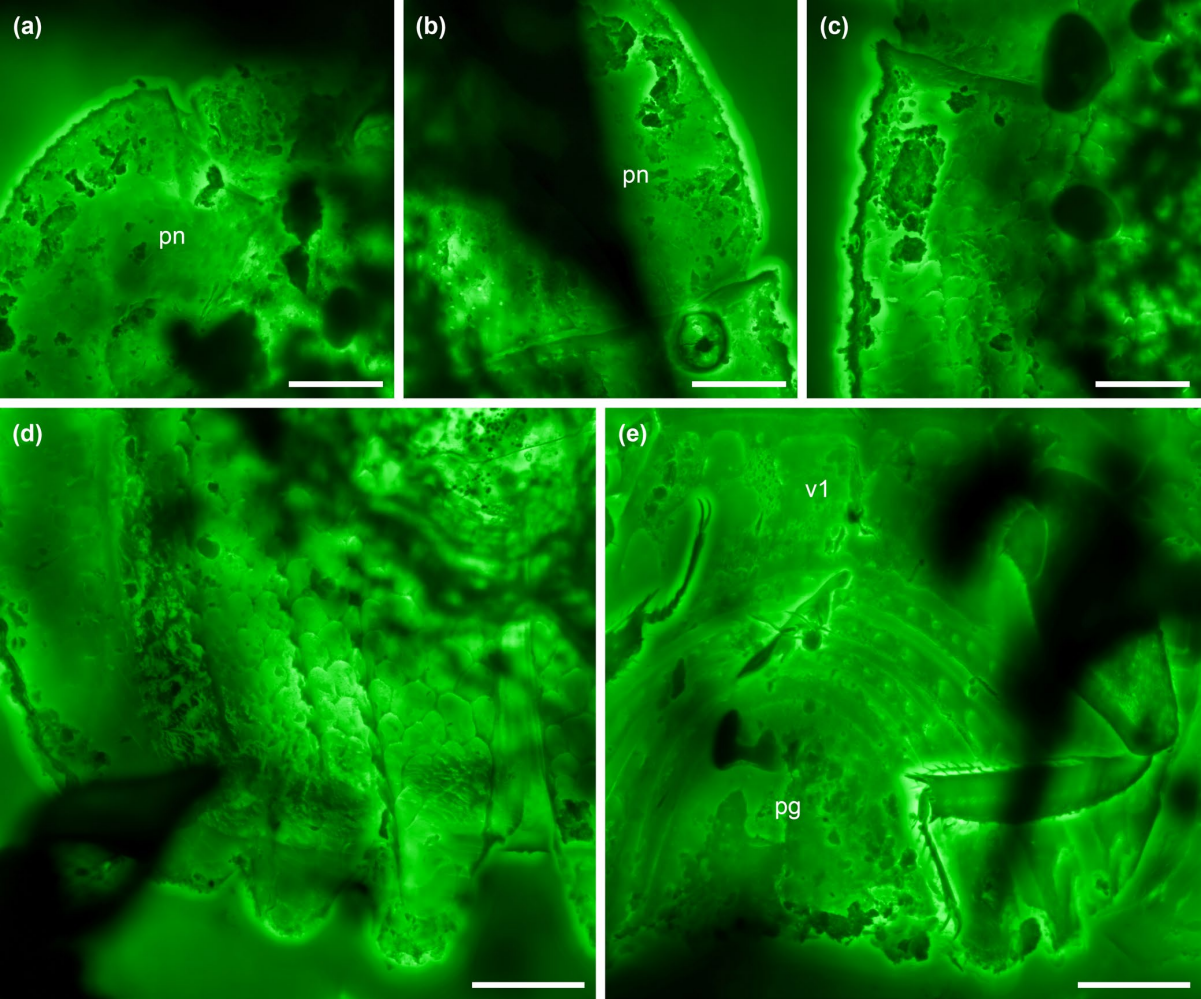
Details of *Fantosmium qizhihaoi*, holotype, NIGP207347, under confocal microscopy. (a, b) Prothorax and head, dorsal view. (c) Elytral base, dorsal view. (d) Elytral apex, dorsal view. (e) Abdomen, ventral view. pg, pygidium; pn, pronotum; v1, abdominal ventrite 1. Scale bars: 200 μm.

The Kachin amber (Burmese amber) specimen studied herein (Figures 1, 2, 3) originated from amber mines near Noije Bum (26°20′N, 96°36′E), Hukawng Valley, Kachin State, northern Myanmar. The amber specimen is deposited in the Nanjing Institute of Geology and Paleontology (NIGP), Chinese Academy of Sciences, Nanjing, China. The amber piece was trimmed with a saw mounted on a handheld rotary tool, ground with emery paper of different grit sizes, and finally polished with polishing powder.

### 2.2 Fossil Imaging

Brightfield images were obtained with a Zeiss Discovery V20 stereo microscope, under reflected illumination. Confocal images were obtained with a Zeiss LSM710 confocal laser scanning microscope, using the 488 nm (Argon) laser excitation line (Fu et al. 2021). Brightfield images were stacked with Zerene Stacker 1.04 and Adobe Photoshop CC. Confocal images were stacked with Helicon Focus 7.0.2 and Adobe Photoshop CC. Images were further processed in Adobe Photoshop CC to adjust brightness and contrast.

### 2.3 Phylogenetic Analyses

**FIGURE 4:**
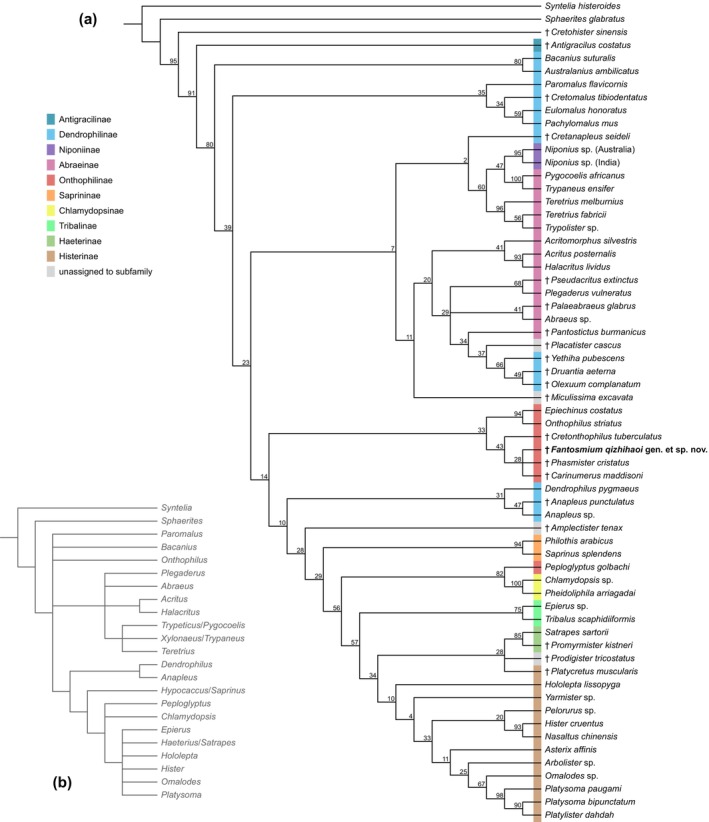
Morphology‐based phylogeny of Histeridae (a), resulting from parsimony analysis under implied weights with molecular‐based backbone (b) as constraints.

The phylogeny of Histeridae cannot be properly resolved based on currently available morphological datasets (Zhou et al. 2020; Simon‐Pražák et al. 2023, 2024). Thus, to evaluate the systematic placement of the new fossil, we conducted a morphology‐based phylogenetic analysis under constrained parsimony (e.g., Li et al. 2023, 2024, 2025). The dataset (File S1) was derived from Simon‐Pražák et al. (2024), which was developed based on Zhou et al. (2020) and Simon‐Pražák et al. (2023). The character list can be found in Zhou et al. (2020). The constraining backbone tree (Figure 4b) was constructed based on the molecular phylogeny of Zhou et al. (2020: figure 2). Since Zhou et al. (2020) used a relatively limited set of genes, we adopted only the clades with a Bayesian posterior probability (BPP) greater than 0.9 (the clade of Histerinae + Haeteriinae + *Epierus* Erichson, although poorly supported in the original analysis by Zhou et al. 2020, was also adopted as it was recovered with BPP > 0.9 in our reanalysis using the site‐heterogeneous CAT‐GTR model).

The analysis was performed under implied weights in R 4.1.0 (R Core Team 2021), using the R script provided by Li et al. (2024) (File S2), which deploys the R package TreeSearch 1.6.0 (Smith 2023). The concavity constant was set to 12, following the suggestion by Goloboff et al. (2018) and Smith (2019). The resulting tree was visualized with the online tool iTOL 7.1 (Letunic and Bork 2024) and graphically edited with Adobe Illustrator CC 2017.

## 3 Systematic Paleontology

Order Coleoptera Linnaeus, 1758.

Family Histeridae Gyllenhal, 1808.

Subfamily Onthophilinae MacLeay, 1819.

### 3.1 Genus *Fantosmium* gen. nov.

**Type species.** *Fantosmium qizhihaoi* sp. nov.

**Etymology.** The generic name is an arbitrary combination of letters inspired by the Greek *phantasma* (ghost), as the shape of this beetle resembles the cartoon depiction of a ghost. The name is neuter in gender.

**Diagnosis.** Clypeus medially longitudinally carinate; carina abruptly expanding anteriorly into triangular plate (Figure 2a). Antennal club with segmental sutures complete (Figure 2c). Pronotum laterally horizontally explanate (Figure 1a). Hypomeral antennal cavity ventrally largely open (Figure 2c). Prosternum distinctly produced medially, forming chin piece (prosternal lobe) (Figure 2a). Elytra each with three carinae; carinae weakly elevated, ornamented with small denticles; intercarinal surface with irregular reticulate sculpture; elytral epipleura horizontally explanate and irregularly punctate; posterior elytral margin strongly undulate (Figure 3c,d). Ventral side of mesothorax, metathorax, and abdominal ventrite 1 with enlarged, irregularly shaped depressions (Figure 2b).

**Remarks.** *Fantosmium* is distinctive among onthophiline genera in possessing a unique combination of elytral features, including weakly elevated carinae ornamented with small denticles, an intercarinal surface with irregular reticulate sculpture, horizontally explanate epipleura, and a strongly undulate posterior elytral margin. These features readily distinguish *Fantosmium* from all other extant and fossil genera of the subfamily.

### 3.2 *Fantosmium qizhihaoi* sp. nov.

**Material.** Holotype, NIGP207347 (Figures 1, 2, 3).

**Etymology.** The species is named after Mr. Zhi‐Hao Qi, who kindly donated the studied specimen.

**Locality and Horizon.** Amber mine located near Noije Bum Village, Tanai Township, Myitkyina District, Kachin State, Myanmar; unnamed horizon, mid‐Cretaceous, Upper Albian to Lower Cenomanian.

**Diagnosis.** As for the genus.

**Description.** Body broadly oval, appearing relatively flattened, about 2.5 mm long, 2.0 mm wide; surface generally without setae or hairs.

Head strongly hypognathous and retracted into prothorax. Clypeus medially longitudinally carinate; carina abruptly expanding anteriorly into triangular plate. Antennal scape elongate; club 3‐segmented with segmental sutures complete.

Pronotum about half as long as wide, widest posteriorly, laterally horizontally explanate; lateral edges slightly curved and anteriorly converging, simple; posterior edge likely weakly angularly projecting; pronotal disc possibly with a pair of short carinae on surface. Hypomeral antennal cavity largely open ventrally, only slightly covered by weak prosternal alae. Prosternum distinctly produced medially, forming chin piece (though without a clear suture separating the so‐called presternum from the rest of prosternum; see Ôhara 1994). Prosternal process complete, moderately wide (seemingly narrower than at least most other Onthophilinae), apically concave. Procoxal cavities strongly transverse, moderately open externally. Elytra subrectangular, about 0.75× as long as their combined width, with anterolateral angles broadly acute, posteriorly overall truncate but markedly undulate (each exhibiting two distinct notches); each elytron with three carinae which are only weakly elevated but ornamented with small denticles; intercarinal surface with irregular reticulate sculpture; elytral epipleura horizontally explanate and irregularly punctate. Mesoventrite short, transverse, with two pairs of depressions. Mesocoxal cavities widely separated. Metaventrite distinctly transverse, with multiple irregularly shaped depressions (not completely symmetrical). Metacoxae widely separated, not extending laterally to meet elytra; plates absent. Protibiae flattened, with weakly setose outer margin; tibial spurs 2‐2‐2, well developed, unequal in length. Tarsi 5‐5‐5; tarsomeres simple. Pretarsal claws simple.

Abdomen with five ventrites. Ventrite 1 considerably longer than subsequent ones, with irregularly shaped depressions; intercoxal process truncate. Ventrites 2–5 very short, strongly curved. Pygidium ventrally oriented, subtriangular to bell‐shaped.

## 4 Discussion

Based on our phylogenetic analysis, *Fantosmium* exhibits affinities with the subfamily Onthophilinae (Figure 4a). The presence of elytral carinae in extant genera such as *Onthophilus* Leach, *Epiechinus* Lewis, and *Peploglyptus* LeConte is also observed in *Fantosmium*, representing a notable morphological resemblance. However, the circumscription of Onthophilinae in extant taxa remains somewhat ambiguous, as different authors have included varying genera within the subfamily (e.g., Kovarik 1994; Ślipinśki and Mazur 1999; Bousquet and Laplante 2006; Kovarik and Caterino 2016). Recent molecular phylogenies further suggest that Onthophilinae *sensu* Kovarik and Caterino (2016) is likely not monophyletic, with *Onthophilus* and *Peploglyptus* appearing distantly related (Zhou et al. 2020).

In extant *Onthophilus* and *Epiechinus*, the anterior margin of the median part of the prosternum and the lateral prosternal alae is approximately straight, and the antennal cavity is mostly closed ventrally (Helava 1978; Ôhara and Nakane 1986; Ôhara 1994; Zhou and Luo 2001; Kovarik and Skelley 2019; Li et al. 2022). In contrast, *Fantosmium* possesses a medially protruding prosternum that forms a distinct chin piece (prosternal lobe), and its antennal cavity is largely open ventrally. In these respects, *Fantosmium* more closely resembles several Cretaceous fossil genera previously assigned to Onthophilinae, namely *Cretonthophilus* Caterino et al., *Phasmister* Caterino, and *Carinumerus* Caterino (Caterino and Maddison 2018; Caterino 2021; Caterino and Yamamoto 2023). According to the hypothesis proposed by Simon‐Pražák et al. (2024), the largely open antennal cavity observed in Cretaceous Onthophilinae represents a plesiomorphic condition, potentially serving as a precursor to the more specialized, closed antennal cavities found in modern *Onthophilus* and *Epiechinus*.

*Fantosmium* can be readily distinguished from other onthophiline genera by a combination of distinctive morphological features. In Cretaceous *Cretonthophilus*, *Phasmister*, and *Carinumerus*, as well as extant *Onthophilus*, the elytral carinae are typically well‐developed and more or less smooth. In contrast, the elytral carinae of *Fantosmium* are quite weak in elevation but ornamented with small denticles. Furthermore, in *Phasmister*, *Carinumerus*, and most extant genera such as *Onthophilus*, *Epiechinus*, *Glymma* Marseul, and *Sigillum* Thérond, the spaces between elytral carinae exhibit regularly and strongly punctate striae (de Marseul 1856; Thérond 1975). Although this pattern might be less apparent in *Cretonthophilus*, based on the description by Caterino and Maddison (2018), it likely possesses foveate striae as well. In *Fantosmium*, however, no regularly punctate striae are observed between the carinae, at least under confocal microscopy. Instead, the intercarinal surface is marked by an irregular reticulate sculpture. In *Peploglyptus*, the punctures on the elytra are somewhat irregular in arrangement, but this condition is still distinct from that of *Fantosmium* and lacks any development of a reticulate sculpture (Caterino 2004). Another diagnostic feature of *Fantosmium* is the presence of horizontally explanate epipleura, which are absent in all known extant and fossil Onthophilinae. These epipleura are punctate, but the punctures are distributed irregularly. In addition, the elytra of *Fantosmium* display a uniquely undulate posterior margin, which further distinguishes it from related taxa.

## Author Contributions

**Yan‐Da Li:** conceptualization (equal), data curation (equal), formal analysis (equal), investigation (equal), visualization (equal), writing – original draft (equal), writing – review and editing (equal). **Di‐Ying Huang:** funding acquisition (equal), investigation (equal), writing – review and editing (equal). **Chen‐Yang Cai:** conceptualization (equal), funding acquisition (equal), investigation (equal), supervision (equal), writing – review and editing (equal).

## Conflicts of Interest

The authors declare no conflicts of interest.

## Supporting information


**File S1:** Morphological data matrix.
**File S2:** R script for constrained parsimony analysis of morphological data.

## Data Availability

The data matrix and R script for the phylogenetic analysis are available in the Supporting Information. The original confocal data are available in the Zenodo repository (https://doi.org/10.5281/zenodo.15730640).
